# Effects of Synthetic Short Cationic Antimicrobial Peptides on the Catalytic Activity of Myeloperoxidase, Reducing Its Oxidative Capacity

**DOI:** 10.3390/antiox11122419

**Published:** 2022-12-07

**Authors:** Tatyana V. Vakhrusheva, Alexey V. Sokolov, Grigoriy D. Moroz, Valeria A. Kostevich, Nikolay P. Gorbunov, Igor P. Smirnov, Ekaterina N. Grafskaia, Ivan A. Latsis, Oleg M. Panasenko, Vassili N. Lazarev

**Affiliations:** 1 Department of Biophysics, Federal Research and Clinical Center of Physical-Chemical Medicine of Federal Medical Biological Agency, 119435 Moscow, Russia; 2 Department of Molecular Genetics, Institute of Experimental Medicine, 197376 St. Petersburg, Russia; 3 Department of Biological and Medical Physics, Moscow Institute of Physics and Technology (State University), Moscow Region, 141701 Dolgoprudny, Russia; 4 Department of Cell Biology, Federal Research and Clinical Center of Physical-Chemical Medicine of Federal Medical Biological Agency, 119435 Moscow, Russia

**Keywords:** synthetic cationic antimicrobial peptides, myeloperoxidase, myeloperoxidase peroxidase activity, myeloperoxidase chlorinating activity, peptide antioxidants

## Abstract

Cationic antimicrobial peptides (CAMPs) have gained attention as promising antimicrobial therapeutics causing lower or no bacterial resistance. Considerable achievements have been made in designing new CAMPs that are highly active as antimicrobials. However, there is a lack of research on their interaction with biologically important proteins. This study focused on CAMPs’ effects on myeloperoxidase (MPO), an enzyme which is microbicidal and concomitantly damaging to host biomolecules and cells due to its ability to produce reactive oxygen and halogen species (ROS/RHS). Four CAMPs designed by us were employed. MPO catalytic activity was assessed by an absorbance spectra analysis and by measuring enzymatic activity using Amplex Red- and Celestine Blue B-based assays. The peptide Hm-AMP2 accelerated MPO turnover. Pept_1545 and Hm-AMP8 inhibited both the MPO chlorinating and peroxidase activities, with components of different inhibition types. Hm-AMP8 was a stronger inhibitor. Its K_i_ towards H_2_O_2_ and Cl^–^ was 0.3–0.4 μM vs. 11–20 μM for pept_1545. Peptide tyrosine and cysteine residues were involved in the mechanisms of the observed effects. The results propose a possible dual role of CAMPs as both antimicrobial agents and agents that downregulate MPO activation, and suggest CAMPs as prototypes for the development of antioxidant compounds to prevent MPO-mediated ROS/RHS overproduction.

## 1. Introduction

The steady increase in and spread of bacterial antibiotic resistance have led to an urgent request for the development of novel antimicrobials. High expectations for combating pathogens are laid on antimicrobial peptides (AMPs) [1,2,3]. The innate immunity of all living organisms from bacteria to animals has been equipped with AMPs [4,5,6]. Among natural AMPs, the vast majority are cationic peptides (CAMPs), with net charges from +2 to +9 (at physiological pH) and length from 10 to 50 amino acid residues with the molecular weight within 10 kDa [7]. In addition to the overall hydrophobicity, CAMPs usually adopt an amphipathic conformation, which facilitates their interaction with negatively charged bacterial cell membranes [8]. The mechanisms of action of CAMPs differ from that of traditional antibiotics which is target-directed and favors the development of antibiotic resistance in bacteria. By contrast, CAMPs have an advantage by aiming at multiple non-specific targets on the plasma membrane and inside the cell, inducing rapid death of bacteria and preventing the emergence of resistance [9,10,11,12]. CAMPs cause cytoplasmic membrane disturbance via electrostatic and hydrophobic interactions dependent on the peptide structure and membrane properties, and it is difficult for bacteria to maintain membrane functional and structural integrity [13]. Specific features of bacterial membranes, such as lipid composition, determine the selectivity of CAMPs for bacterial cells vs. mammalian cells [14,15,16].

In searching for therapeutic CAMPs, priority has been given to synthetic CAMPs with short molecular length (less than 30 amino acid residues) due to relatively simple and low-cost production [17]. With possibilities of novel methods and technologies, including machine learning, it has become possible to predict peptide sequences with antimicrobial activity [18,19,20]. Substantial progress has been made in design and synthesis of novel CAMPs, improving the biological activities of the natural analogs, such as efficacy, selectivity, and stability [21,22]. When developing CAMPs and evaluating their biocompatibility, attention has been given to the analysis of their cytotoxic action. There are only a few studies on the interaction of CAMPs with biologically important proteins.

Our recent research demonstrated that CAMP-induced hemolysis was reduced by human serum albumin, presumably through CAMP-albumin binding that prevented translocation onto cells [23]. CAMP binding to human or bovine serum albumin has also been reported by [24,25]. Our previous data showing CAMP-induced prolongation of plasma coagulation time indicated the interaction of CAMPs with coagulation factors [23]. Short non-helical CAMPs have been found to interact with ATP and inhibit certain ATP-dependent enzymes such as luciferase, DnaK, and DNA polymerase [26].

Our present work focuses on the interaction of CAMPs with myeloperoxidase (MPO), an enzyme involved in oxygen-dependent neutrophil antimicrobial activity. The co-localization of CAMPs and MPO is proposed to occur at infected sites, to which neutrophils are recruited and where CAMPs should be directed. Neutrophils, the effector cells in both innate and adaptive immunity, are part of the body’s first line of defense against pathogens. They rapidly move out of blood vessels to sites of infection, guided by chemotactic factors, and display an impressive arsenal of mechanisms to destroy the invading microbes. The antimicrobial action of neutrophils is provided largely by enzymes produced during granulopoiesis and stored in cytosolic granules [27]. Upon neutrophil activation, degranulation occurs, resulting in the release of these enzymes into the phagosome and also partially into the extracellular milieu, where they are in a free state or bound on neutrophil extracellular traps.

MPO, a heme protein, is expressed mainly in neutrophils and to a lesser extent in monocytes and tissue macrophages. In the resting cell, it is located in azurophilic granules. MPO plays a key role in host defense by destroying pathogens, due to its unique ability to produce hypochlorous acid (HOCl), a highly reactive oxidizing compound. In addition to HOCl, a number of other compounds capable of bactericidal action are formed in MPO-mediated reactions. However, due to their high reactivity, HOCl and other MPO products can also target biologically important molecules (proteins, lipids, and DNA), cells, and tissues of the host [28]. As a result, MPO is implicated in many pathophysiological conditions and diseases associated with inflammation (cardiovascular and neurodegenerative diseases, asthma, diabetes, cancer, etc.). Thus, studies on the effects of different compounds on MPO catalytic activity are of great importance.

A simplified scheme for reactions in MPO catalytic cycles is as follows [29,30]:

MPO (Fe^3+^) + H_2_O_2_ → Compound I (O=Fe^4+•^) + H_2_O

Compound I (O=Fe^4+•^) + AH_2_ → Compound II (O=Fe^4+^) + AH^•^

Compound II (O=Fe^4+^) + AH_2_ → MPO (Fe^3+^) + AH^•^ + H_2_O

                            (Peroxidase cycle)

MPO (Fe^3+^) + H_2_O_2_ → Compound I (O=Fe^4+•^) + H_2_O

Compound I (O=Fe^4+•^) + X^−^ + H^+^ → MPO (Fe^3+^) + HOX + H_2_O,

                           where X = Cl^−^, Br^−^, I^−^, SCN^−^.

                           (Halogenation cycle)

Unlike other peroxidases, MPO can pass through the classical peroxidase cycle and also the so-called halogenation cycle. While MPO follows the peroxidase cycle, a two-electron oxidation of native, ferric form of MPO (Fe^3+^) to Compound I is followed by two successive one-electron reductions back to native enzyme via Compound II. In the halogenation cycle, Compound I is directly reduced back to the resting state by halide (Cl^−^, Br^−^, I^−^) or pseudohalide (SCN^−^) anions to form hypo(pseudo)halous acids (HOCl, HOBr, HOI, or HOSCN). The native MPO and its intermediate redox forms have distinct absorbance spectra, which makes spectrum analysis a convenient tool for studying MPO catalytic activity.

In regard to the effects of CAMPs on MPO, it has been shown that a synthetic CAMP derived from the N terminus of human lactoferrin inhibited MPO [31]. In this study, we analyzed the interaction of MPO with four CAMPs designed by us earlier [20,32]. The peptides had diverse spatial structures, and differed in length, charge, and other characteristics. We examined the influence of CAMPs on MPO catalytic activity by monitoring peptide-induced changes in absorbance spectra of the MPO heme during the H_2_O_2_-inintiated peroxidase reaction and by assessing MPO peroxidase activity as well as chlorinating activity using an Amplex Red-based and Celestine Blue B-based assay, respectively. In order to spawn an explanation of the results, experiments were carried out on the involvement of redox-active amino acid residues of the peptides in mechanisms of the observed effects. Novel data on the interaction of peptide-linked tyrosine and cysteine with MPO were obtained. The study showed that short CAMPs, depending on their structure, are able to interact with MPO, significantly modulating its catalytic activity through different mechanisms. In the process of elucidating the interaction of peptides with H_2_O_2_-activated MPO, a hypothesis for the role of peptides as MPO substrates arose, which we tested. The results propose that CAMPs inhibiting MPO could serve as prototypes for the development of antioxidant agents to downregulate MPO in inflammation featured by oxidative stress resulting from MPO-mediated overproduction of reactive oxygen and halogen species (ROS/RHS).

## 2. Materials and Methods

### 2.1. Reagents

Phosphate buffered saline (PBS) tablets, sodium phosphate monohydrate NaH_2_PO_4_*H_2_O, potassium phosphate KH_2_PO_4_, sodium chloride NaCl, potassium iodide KI, sodium acetate trihydrate CH_3_COONa*3H_2_O, taurine, 30% hydrogen peroxide (H_2_O_2_) solution, sodium hypochlorite solution (with 4.00–4.99% available chlorine), 5,5′-dithio-*bis*-(2-nitrobenzoic acid) (DTNB), *d*iethylenetriaminepentaacetic acid (DTPA), formic acid, tris hydrochloride (Tris-HCl), Celestine Blue B (CB), and Amplex Red™ Reagent (10-acetyl-3,7-dihydroxyphenoxazine (ADHP)) were purchased from Sigma-Aldrich (Sigma-Aldrich, St. Louis, MO, USA).

Synthesis of peptides was carried out by solid-phase peptide synthesis in a Liberty Blue automated microwave peptide synthesizer (CEM Corporation) by using Rink Amide NovaGel Novabiochem (0.69 mM/g) as the solid phase [20,32]. Fmoc-protected amino acid derivatives from Sigma-Aldrich were applied in the synthesis. Preparative purification of synthesized products was carried out by reversed-phase chromatography in H_2_O/AcN gradient. Peptide purity was verified by HPLC-MS. ZORBAX SB-C18 chromatography columns (Agilent Technologies, Santa Clara, CA, USA) were used for quantitative chromatographic analysis and confirmation of peptide purity. All peptides were >95% pure.

H_2_O_2_ working solutions were prepared freshly by dilution of the commercial solution. The H_2_O_2_ concentration was measured by absorbance at 240 nm (ε_240_ = 43.6 M^−1^ cm^–1^) [33].

The experiments were carried out at room temperature, unless indicated otherwise.

### 2.2. Myeloperoxidase

Native myeloperoxidase (MPO) was obtained from human leukocyte extract by successive chromatography on heparin-Sepharose and phenyl-agarose followed by gel-filtration on a Sephacryl S-200 HR, as previously described [34]. The Reinheit Zahl value (A_430 nm_/A_280 nm_) for the purified protein was 0.85. The MPO concentration was determined as the heme concentration measured spectrophotometrically at 430 nm with a molar extinction coefficient of 91,000 M^−1^ cm^−1^ [35].

### 2.3. Absorbance Spectra

Spectra of MPO were recorded on a Cary 50 Bio UV-Vis spectrophotometer (Varian, Mulgrave, Australia). Experiments were performed with Eppendorf UVette plastic UV/Vis cuvettes (Hamburg, Germany). An MPO concentration was 2 μM heme. MPO was mixed with CAMP in 10 mM Na-phosphate buffer (pH 7.4) supplemented with 0.2 mM DTPA. H_2_O_2_ was added as a microvolume of concentrated solution (100 μM final).

### 2.4. Dityrosine Fluorescence Detection

Formation of dityrosine during the MPO reaction with CAMPs was monitored by fluorescence on a CM 2203 spectrofluorometer (Minsk, Belarus) with an excitation wavelength of 315 nm. Emission spectra were recorded between 350 nm and 500 nm, with the maximum being at 405–410 nm [36,37]. MPO (0.4 μM) in a 1 mL cuvette was mixed with CAMP (40 μM) at pH 7.4 in 10 mM Na-phosphate buffer supplemented with 0.2 mM DTPA, and the starting emission spectrum was recorded. Succeeding spectra were taken at time intervals following the addition of 50 μM H_2_O_2_ to the continuously stirred reaction mixture.

### 2.5. Mass Spectrometry

Mass spectra were acquired using an Orbitrap Q Exactive HF-X mass spectrometer equipped with HESI source in the positive ion mode (Thermo Fisher Scientific, Waltham, MA, USA). Direct infusion syringe pump sampling was carried out with a 100 mL Hamilton syringe at a flow rate of 5 mL/min. The spray voltage was set at +3.5 kV, the sheath and auxiliary gas flow rate at 5 and 1, respectively; the S-lens RF level at 65; the capillary temperature at 250 °C. The resolution was set at 240,000, the AGC target at 1E6, and the maximum injection time at 50 ms. A summary spectrum was collected over 4 min and used for calculating S/N and measuring peak intensities. Before analysis, desalting of samples was carried out using C-18 ZIP-TIP (Millipore, Billerica, MA, USA), according to the manufacturer’s instructions. The eluate was mixed with 0.1% formic acid in DI water (18 MOhm) to a volume of 100 μL. The solutions prepared were used for direct infusion.

### 2.6. Measurement of Sulfhydryl Groups

The peroxidase reaction was carried out in 10 mM Na-phosphate buffer, pH 7.4 (containing 0.2 mM DTPA), in a volume of 60 μl placed in the wells of a 96-well plate. Peptides were added to MPO (1 μM heme) to a concentration of 150 μM. Following 5 min of incubation, H_2_O_2_ (100 μM, final) was added to test samples to trigger the reaction. Control samples were the same except H_2_O_2_. The content of free sulfhydryl groups was estimated spectrophotometrically by using 5,5′-dithiobis(2-nitrobenzoic acid) (DTNB, Ellman’s reagent) prepared in 0.4 M Tris-HCl buffer, pH 9.0. At a desired time after the reaction was started, 120 μL aliquots of freshly prepared DTNB-containing buffer were added to one part of samples (160 μM DTNB, final) and 160 μL of the buffer without DTNB was added to another part of the same samples. Measurements were made with a Multiskan Ascent plate reader (Thermo Electron Corporation, Vantaa, Finland). Absorbance in the absence of DTNB was subtracted from that in the presence of DTNB. The loss of sulfhydryl groups was expressed as a percentage relative to controls incubated without H_2_O_2_.

### 2.7. Myeloperoxidase Peroxidase Activity

MPO peroxidase activity was evaluated fluorometrically using Amplex Red^TM^ reagent which was oxidized by MPO to the fluorescent resorufin (λ_ex_ = 530 nm, λ_em_ = 590 nm) [38]. The assay was performed in 96-plate format. The final reaction mixture in 50 mM K-phosphate buffer (pH 7.4) was as follows: MPO, 8 nM heme; peptide, 0.5–8.0 μM; H_2_O_2_, 25 μM; Amplex Red reagent, 50 μM. MPO was preincubated with peptides for 5 min in a volume of 100 μl. Then, Amplex Red was added, and the enzymatic reaction was initiated by adding H_2_O_2_ (a final probe volume of 200 μL per well). The fluorescence measurement was made with a CLARIOstar multimodal plate reader (BMG Labtech, Ortenberg, Germany) at 5 min after the beginning of the reaction. To determine the type of CAMP-induced inhibition, K_M_ and V_max_ were obtained by varying substrates of MPO: Amplex Red in a final concentration from 1 to 62.5 μM, or H_2_O_2_ in a final concentration from 0.625 to 20 μM.

### 2.8. Myeloperoxidase Chlorinating Activity

MPO chlorinating activity was evaluated fluorometrically using Celestine Blue B (CB) which was oxidized to fluorescent glycol (λ_ex_ = 487 nm, λ_em_ = 578 nm) by taurine N-chloramine produced in the reaction of taurine with HOCl generated by MPO [39]. The assay was performed in 96-plate format. MPO was preincubated with peptides for 5 min in a volume of 100 μL, with the concentrations being: MPO, 1.6 nM heme and peptide, 16 nM–16 μM. Then, 100 μL aliquots of CB-containing mix (CB, 400 µM; H_2_O_2_, 100 µM; taurine, 4 mM; KI, 10 µM; NaCl, 300 mM; 50 mM Na-acetate buffer, pH 5.5) were added to MPO/peptide and to 100 μL of HOCl standards (0–150 μM). The fluorescence measurement was made with a CLARIOstar multimodal plate reader (BMG Labtech, Ortenberg, Germany) at 15 min after the reaction started. Fluorescence intensity detected for HOCl standards of HOCl was used for calculating the chlorinating activity as mole of HOCl per mole of MPO heme per second. To determine the type of CAMP-induced inhibition, K_M_ and V_max_ were obtained by varying substrates of MPO: NaCl in a final concentration from 25 to 300 mM, or H_2_O_2_ in a final concentration from 5 to 80 µM.

### 2.9. Calculation of Michaelis Constants, Maximal Rate, and Inhibition Constants

The Hannes-Wolf plots were made as ([Substrate]/V = f([Substrate]). K_M_ was equal in magnitude to the intercept made on the abscissa axis by the approximating straight line; V_max_ was calculated as a reciprocal value of the straight-line slope. The inhibition constant (K_i_) for noncompetitive inhibition was calculated as follows: K_i_ = [I]/((V_max_ − V_max_’)/V_max_’); K_i_ for competitive inhibition was calculated as follows: K_i_ = [I]/((K_M_’ − K_M_)/K_M_); K_i_ for mixed inhibition was calculated as follows: K_i_ = [I]/(((K_M_’ − K_M_)/K_M_)^2^ + ((V_max_ − V_max_’)/V_max_’))^2^)^0.5^; and K_i_ for uncompetitive inhibition was calculated as follows: Ki = [I]/(((K_M_ − K_M_’)/K_M_’)^2^ + ((V_max_ – V_max_’)/V_max_’))^2^)^0.5^, where [I]—concentration of inhibitor (CAMP), K_M_—Michaelis constant without inhibitor, V_max_—maximal rate without inhibitor, K_M_’—Michaelis constant in the presence of inhibitor, V_max_’—maximal rate in the presence of inhibitor.

### 2.10. Data Analysis

Data are expressed as mean ± SD of three independent experiments with triplicate or duplicate samples in each experiment. The statistical analysis was carried out by Student’s *t*-test to determine a significant difference between the control samples and the others. A *p*-value <0.05 was considered to be statistically significant.

## 3. Results

### 3.1. Characteristics of CAMPs Used in This Study

CAMPs were designed and synthesized using bioinformatic analysis of the medicinal leech microbiome, as described previously in our works [20,32]. Their amino acid sequences, molecular masses, charges, etc. are provided in Table 1.

For characteristics describing the antibacterial and hemolytic activities of CAMPs under study see Table S1 and Figure S1.

### 3.2. Detection of CAMP Effects on MPO Catalytic Activity Using Absorbance Spectra

To elucidate the influence of CAMPs on MPO catalytic activity, we analyzed absorbance spectral changes for MPO activated by H_2_O_2_ in the presence of peptides. The spectra were obtained with 2 μM MPO heme and 100 μM H_2_O_2_ in 10 mM Na-phosphate buffer, pH 7.4. All reactions in these and further experiments were performed in buffers supplemented with 0.2 mM diethylenetriaminepentaacetic acid (DTPA) to sequester free transition metal ions to prevent tyrosine and cysteine autooxidation.

**Figure 1:**
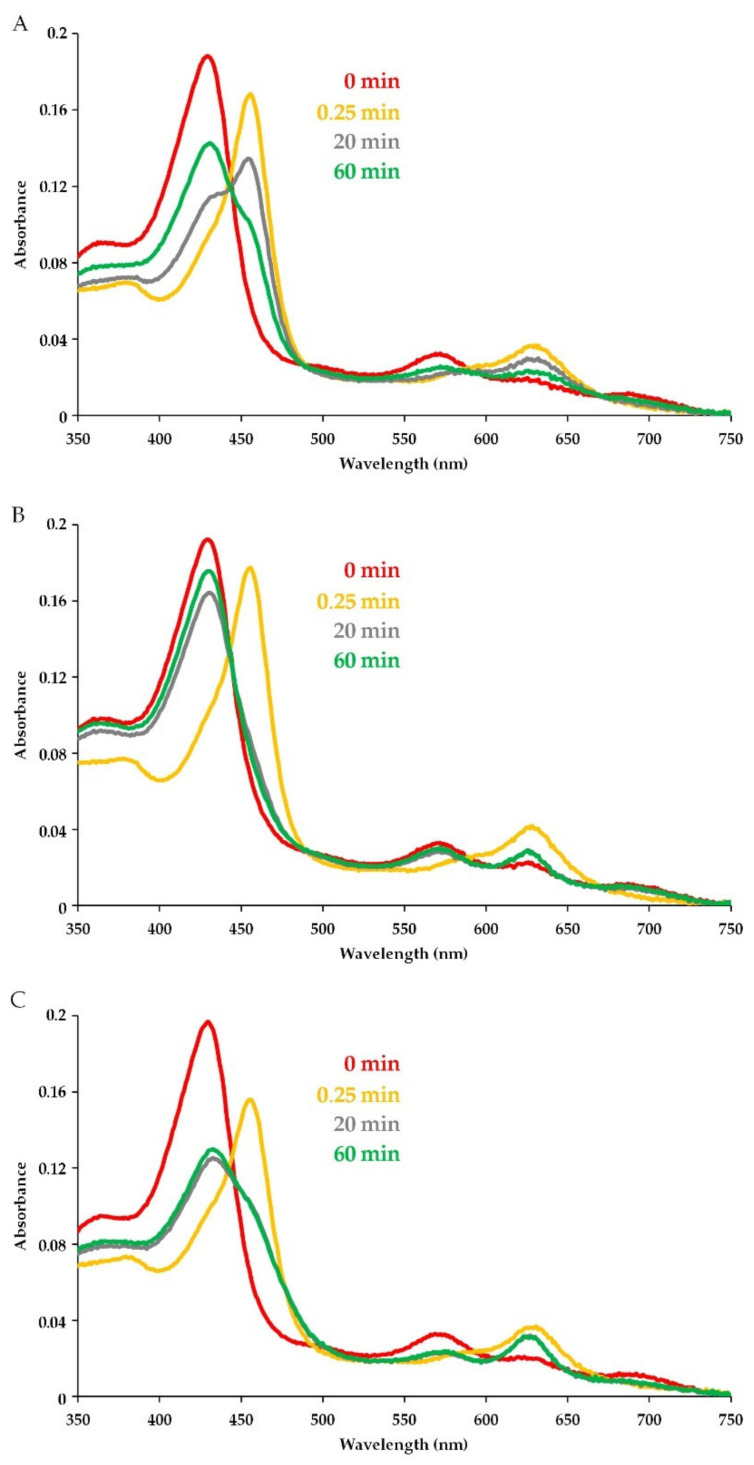
Illustrative absorbance spectra reflecting the reaction of myeloperoxidase (MPO, 2 µM heme) with H_2_O_2_ (100 µM) in the absence and presence of cationic antimicrobial peptides (CAMPs) in 10 mM Na-phosphate buffer, pH 7.4: (**A**) MPO in the absence of peptides; (**B**) peptide-induced accelerating effect (on the example of pept_1545 at a concentration of 150 μM); (**C**) peptide-induced inhibitory effect (on the example of Hm-AMP8 at a concentration of 150 μM). Spectra were taken before (0 min, red color) and 0.25, 20, and 60 min after adding H_2_O_2_.

The absorbance spectra of MPO upon reaction with H_2_O_2_ in the absence of peptides are shown in Figure 1A. The starting spectrum (0 min in Figure 1) was characteristic of ferric MPO (Fe^3+^), with a Soret band at 430 nm and a band at 570 nm. Adding H_2_O_2_ caused a quick decline in absorbance at 430 nm and 570 nm and the appearance of absorbance peaks at 455 nm and 630 nm, which is indicative of transition to Compound II, an inactive MPO intermediate. Compound II formation was followed by the gradual decay to the native enzyme (Figure 1A). The highly reactive Compound I, which is formed at the first step of MPO turnover, is known to be rapidly reduced (in milliseconds) by H_2_O_2_ (or another electron donor) to Compound II [29,41,42]. Compound II is more stable, and its reduction to the native MPO is slower. In addition, a 50-fold excess of H_2_O_2_ over heme (as it was in our experiments) allows obtaining a relatively long-living Compound II [42,43,44,45].

The MPO absorbance spectrum was not changed by peptides before H_2_O_2_ addition (Figure 1A–C, red color). However, after H_2_O_2_ was added to trigger the enzymatic reaction, the pattern of absorbance changes in the presence of peptides differed from that observed for MPO alone. When comparing spectra in Figure 1B to those in Figure 1A, it is apparent that pept_1545 at a concentration of 150 μM accelerated the enzyme turnover. By contrast, peptide Hm-AMP8 blocked the completion of Compound II decay and the enzyme return to the resting state (Figure 1C).

**Figure 2:**
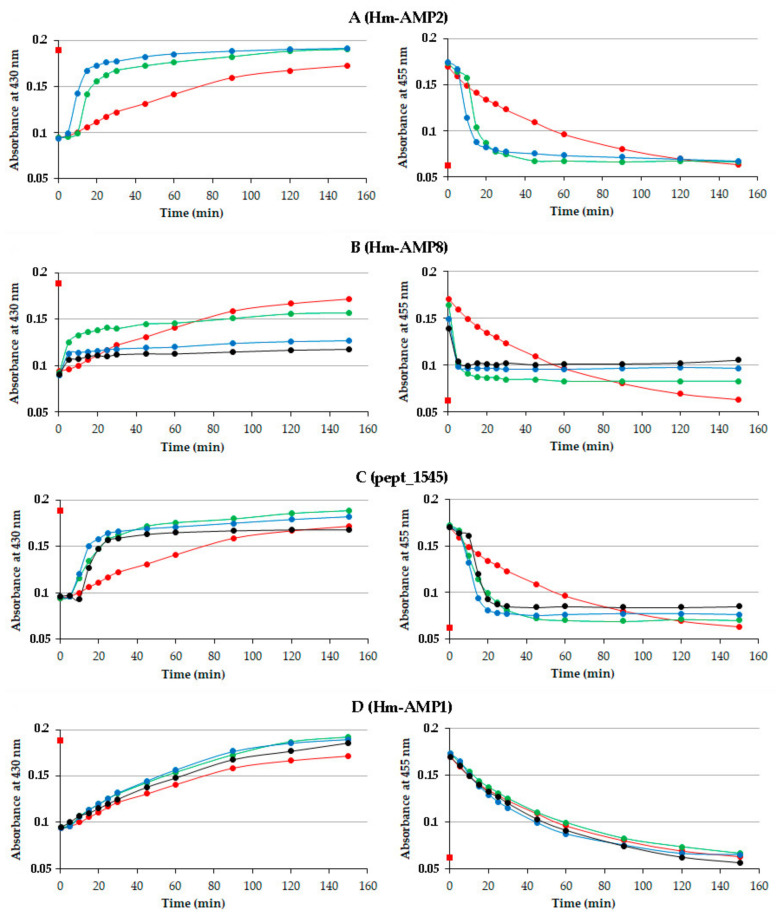
Time courses of spectral absorbance changes at 430 nm (indicative of MPO ground state) and 455 nm (indicative of Compound II) in the absence and presence of cationic antimicrobial peptides (CAMPs) in the system MPO/H_2_O_2_. The name of CAMP is given in parentheses after the panel designation (**A**–**D**). H_2_O_2_ (100 µM) was added to MPO (2 µM heme) or MPO preincubated with peptides at different peptide/heme molar ratios for 5 min in 10 mM Na-phosphate buffer, pH 7.4. Spectra were taken before and 0.25 min, 5 min, 10 min, and so on after adding H_2_O_2_. The initial absorbance values (before H_2_O_2_ addition) are shown as a red square marker. Red curve—MPO in the absence of peptides; green curve—peptide/heme ratio of 15 mol/mol; blue curve 3—peptide/heme ratio of 75 mol/mol, except for Hm-AMP2 for which it was 45 mol/mol; black curve—peptide/heme ratio of 100 mol/mol for Hm-AMP8 or 100 mol/mol and 125 mol/mol for pept_1545. Data are representative of three independent experiments.

The time course of MPO reaction was monitored by the absorbance changes at 430 nm (native MPO) and 455 nm (Compound II) (Figure 2). The addition of H_2_O_2_ induced an initial rapid drop in the absorbance at 430 nm and an accompanying increase in the absorbance at 455 nm regardless of the presence of CAMPs, indicating than none of CAMPs prevented the formation of Compound I and its conversion to Compound II. Further peptide-induced changes in absorbance spectra varied among CAMPs.

As seen from Figure 2A, Hm-AMP2 accelerated Compound II decay to native MPO, with the effect being greater at a higher peptide/heme molar ratio. Because of poor solubility of Hm-AMP2 in phosphate buffer, the highest peptide/heme molar ratio used was 45:1. The decrease in absorbance at 455 nm was mirrored by the increase in absorbance at 430 nm. The 430 nm and 455 nm absorbances in the presence of Hm-AMP2 both attained the initial values within less time than observed for MPO alone. The results indicate that Hm-AMP2 is capable of increasing the rate of MPO turnover.

Hm-AMP8 caused a marked inhibition of MPO catalytic activity (Figure 1C and Figure 2B). Though, as compared with the control MPO, a faster decrease in absorbance at 455 nm was observed, the absorbance ultimately did not return to the initial level, indicating accumulation of Compound II. Only about half of MPO molecules converted back into the ground state at an Hm-AMP8 concentration of 30 μM (a peptide/heme molar ratio of 15:1), as indicated by absorbance changes at 430 nm. An increase in peptide concentration to 150 μM further decreased the return of the enzyme to its active ferric state. Increasing Hm-AMP8 concentration to 200 μM had no additional effect.

Pept_1545 exhibited a dual effect on MPO activity (Figure 2C). At concentrations of 30 μM and 150 μM, an acceleration of Compound II reduction to ferric MPO was a dominating effect. At concentrations of 200 μM and 250 μM, a 10 min delay in Compound II reduction and MPO return to the native state was observed, indicating that enzyme turnover was reduced. This was followed by a sharp decline in Compound II absorbance along with an increase in ferric MPO absorbance. Finally, however, the Compound II reduction was not completed, which was manifested by an increased absorbance level at 455 nm vs. the initial absorbance or absorbance of control MPO. Thus, the analysis of absorbance spectra of MPO-peptide interactions suggested that Hm-AMP8 and pept_1545 were capable of inhibiting the catalytic activity of MPO, with pept_1545 being less efficient.

Hm-AMP1 had no significant effect on MPO cycling (Figure 2D), with both 430 nm and 455 nm absorbances changing gradually in a similar manner as for control MPO.

The kinetic analysis of the absorbance changes for each of Hm-AMP2, Hm-AMP8, and pept_1545 revealed an initial abrupt increase/decrease in absorbance, which was absent in the gradual absorbance curve for the control MPO. A likely explanation for the peptide-induced acceleration of MPO turnover can be as follows: When Compound II is formed upon excess H_2_O_2_ (as was in our study), its slow conversion to ferric MPO can be accelerated by the reducing substrate additionally present in the reaction mixture. Thus, the data obtained in a broad range of peptide concentrations of 15:1 to 125:1 molar ratio of peptide to enzyme indicate that Hm-AMP2, Hm-AMP8, and pept_1545 could be peroxidase substrates for MPO. This draws attention to the presence of tyrosine and cysteine residues in these peptides.

Free tyrosine is known to be a peroxidase substrate, reacting rapidly with both Compounds I and II. Bimolecular rate constants for reactions of free tyrosine with Compound I and Compound II are 7.7 × 10^5^ M^−1^ s^−1^ and 1.6 × 10^4^ M^−1^ s^−1^, respectively [42]. As a result of these reactions, tyrosine accelerates MPO turnover, thereby, increasing the enzyme catalytic activity. MPO-catalyzed oxidation of tyrosine occurs in the absence as well as presence of chloride, which is accompanied by hastened HOCl production [42,46]. A few studies have shown that peptide-linked tyrosine can also be a substrate for MPO and other peroxidases [47,48,49].

Free cysteine is a poor peroxidase substrate. Poor peroxidase substrates react with Compound I but hardly or not with Compound II and, thereby, could trap the enzyme as Compound II, arresting the catalytic cycle. Bimolecular rate constants for reactions of free cysteine with Compound I and Compound II are 4.1 × 10^3^ M^−1^ s^−1^ and <1 × 10^1^ M^−1^ s^−1^, respectively [50]. Nevertheless, sulfhydryl groups in a number of low-molecular compounds have been shown to react with both Compound I and Compound II, though with restricted structural requirements to be electron donors for MPO [50].

No potential peroxidase substrates are among amino acids of Hm-AMP1.

To react with MPO, a peptide residue has to closely approach to the active site. The active site heme group of MPO is located in a crevice (~15 Å in depth) with a channel (~10 Å in diameter) which opens into the distal cavity [51]. Only H_2_O_2_ and small anions have ready access to the iron atom [52]. Other compounds that are oxidized by MPO via the peroxidase cycle bind in the binding site at the entrance to the distal cavity [53]. The overall hydrophobic character of this binding site may promote interaction with CAMPs possessing hydrophobicity and/or amphipathicity. Despite concerns raised about the accessibility of the MPO active site for peptides longer than tripeptide [54], computational docking results have demonstrated the binding of the peptide GRRRRSVQWCA to the edge of and within the cleft of the active site [31].

Thus, there is reason to believe that Hm-AMP2, Hm-AMP8, and pept_1545 are able to bind to the heme binding pocket, orienting the reactive amino acids to the active site. To clarify the mechanisms of the observed effects of CAMPs, experiments were carried out to detect the products of peptide oxidation by MPO.

### 3.3. Involvement of Peptide Tyrosine Residues in MPO Catalysis

One-electron oxidation of tyrosine by Compound I or II involves the abstraction of hydrogen from tyrosine to yield a tyrosyl radical (Tyr^•^). Tyrosyl radicals are unstable and readily combine to form *o,o*′-dityrosine: 2Tyr^•^→ diTyr. Dityrosine formation has been shown to accompany the oxidation of free tyrosine by cell-free or neutrophil- and macrophage-associated MPO activated by H_2_O_2_ [55,56]. The research by M. Tien demonstrated that tyrosine incorporated into a dipeptide, tripeptide, or pentapeptide retained the ability to react with Compounds I and II with the subsequent formation of dityrosine, but at lower rates as compared with free tyrosine [47]. The rates decreased with increasing peptide size. Peroxidase-induced polymerization of tyrosine-containing peptides through initial formation of an intermediate tyrosyl radical has been substantiated in the work by C. Steffensen et al. [49].

To find out whether tyrosine of Hm-AMP2 and Hm-AMP8 could be oxidized by MPO/H_2_O_2_, we aimed to identify dityrosine as a marker of tyrosine oxidation.

#### 3.3.1. Detection of MPO-Induced Oxidation of Peptide Tyrosine Residues Using Fluorometry

**Figure 3:**
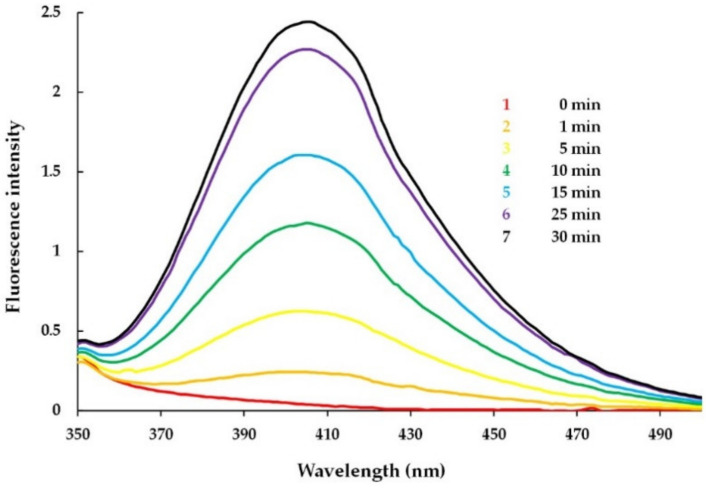
Fluorescence spectra of dityrosine formed during incubation of Hm-AMP2 (40 μM) with MPO (0.4 μM heme) activated by 50 μM H_2_O_2_ in 10 mM Na-phosphate buffer, pH 7.4. Spectra (λ_ex_ = 315 nm) were taken before (0 min, red line) and after adding H_2_O_2_ at time intervals indicated in the figure.

Since dityrosine is an intensely fluorescent compound, the fluorescence method can be applied to detect its formation. MPO (0.4 µM heme) and tyrosine-containing Hm-AMP2 (40 µM) or HM-AMP8 (40 µM) were mixed in a cuvette in 10 mM Na-phosphate buffer, pH 7.4. No emission peak characteristic of dityrosine was observed. Succeeding spectra were recorded at time intervals after H_2_O_2_ (50 µM) addition. Dityrosine formation was monitored by the increase in fluorescence intensity. The results obtained for Hm-AMP2 distinctly indicated the time-dependent generation of dityrosine (Figure 3).

The fluorescence spectra of the reaction mixture of MPO/H_2_O_2_ and Hm-AMP8 showed no reliable presence of dityrosine (data not shown). No dityrosine production is not sufficient to rule out MPO-catalyzed oxidation of tyrosine in Hm-AMP8. The limiting factor could have been dityrosine formation. In a study by H. Zhang and colleagues, a comparison of the oxidation products of N-acetyl lysyltyrosylcysteine amide (KYC) and N-acetyl lysyltyrosylserine amide (KYS) in the MPO/H_2_O_2_ system revealed the formation of dityrosine only by KYS [57]. The experiments demonstrated that MPO oxidized tyrosine residues in both KYC and KYS, but the cysteine in KYC rapidly scavenged tyrosyl radical. It can be proposed that the same was possibly occurring for Hm-AMP8.

Thus, the results of fluorescence experiments supported the interaction between the tyrosine residue of Hm-AMP2 and the MPO active site to produce a tyrosyl radical.

#### 3.3.2. Detection of MPO-Induced Oxidation of Peptide Tyrosine Residues Using Mass Spectrometry

Additional proof for dityrosine formation upon incubation of Hm-AMP2 with H_2_O_2_-activated MPO was obtained by electrospray mass spectrometry which allowed the detection of a peptide product of the molecular weight of the dityrosine cross-linked peptide dimer. Dimerization of peptides via initial formation of a tyrosyl radical results in a 2 Da decrease in the double mass of the peptide to [2M − 2H]. Hence, the dimer sought has a mass of 3454.1 Da.

**Figure 4:**
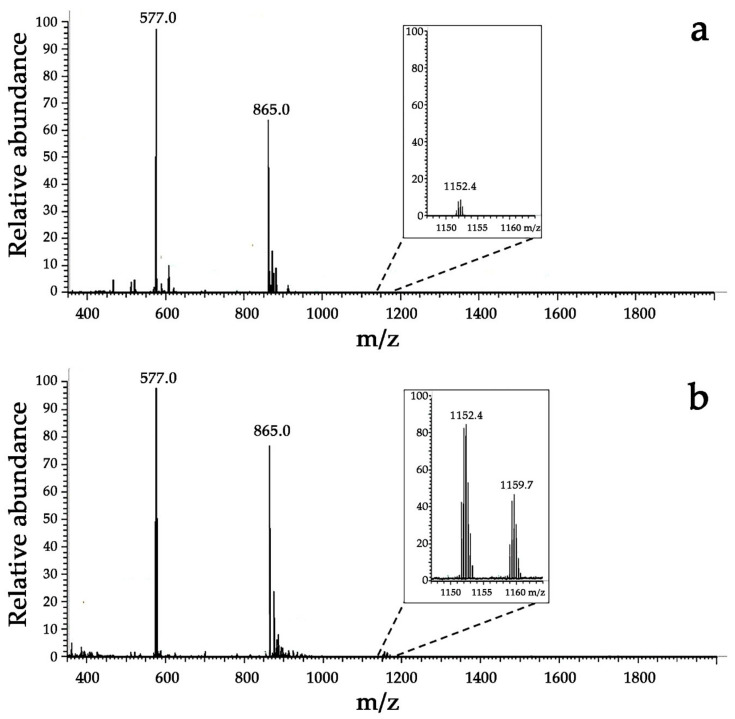
Electrospray mass spectra (in positive mode) of control Hm-AMP2 (**a**) or Hm-AMP2 treated with MPO/H_2_O_2_ (**b**) in 10 mM Na-phosphate buffer, pH 7. The concentrations were 40 μM Hm-AMP2, 0.4 μM MPO heme, and 50 μM H_2_O_2_. Peaks at *m*/*z* = 577.0 and 865.0 are assigned to the peptide’s triply and doubly charged ions, respectively. Enlargement of the peaks at *m*/*z* = 1152.4 and *m*/*z* = 1159.7 in (**b**) (inset) vs. the peaks in (**a**) (inset) shows dityrosine cross-linked peptide dimer resulted from the MPO-mediated reaction.

Mass spectra are shown in Figure 4. A mass spectrum of control Hm-AMP2 sample gave a prominent peak at *m*/*z* = 577.0 which corresponded to the peptide’s triply charged ion with a mass of *m* = [M + 3H]^3+^ (z = 3, spectral *m*/*z* value = 577.0). Another intense peak at *m*/*z* of 865.0 was assigned to the doubly charged peptide ion. Trace amounts of a compound at *m*/*z* = 1152.4 (expanded in the inset in Figure 4a), which may be ascribed to the peptide’s double mass minus two, were also observed. This product probably originated from spontaneous cross-linking reactions. For the Hm-AMP2/MPO/H_2_O_2_ sample in which the presence of dityrosine was fluorometrically confirmed, the intensity of the [2M − 2H + 3H]^3+^ ion signal at *m*/*z* = 1152.4 was found to be 20-fold increased as compared with that in the control spectrum. The signal of the dimerized peptide’s sodium adduct at *m*/*z* = 1159.7 was also observed. These results can be interpreted as an indication of the presence of dityrosine cross-linked Hm-AMP2 molecules. These data, together with fluorescence data, are consistent with the suggestion that tyrosine of Hm-AMP2 is capable of reacting with Compounds I and II with subsequent formation of dityrosine cross-linked peptide dimer.

### 3.4. Involvement of Peptide Cysteine Residues in the Effects of Peptides on MPO Activity

To determine an estimation of whether the cysteine residue of pept_1545 and Hm-AMP8 participated in MPO-mediated reactions, we compared the content of free sulfhydryl groups in the reaction mixtures of peptides (150 μM) with the MPO/H_2_O_2_ system (1 μM heme, 100 μM H_2_O_2_) and control samples containing peptides plus MPO with no added H_2_O_2_. Measurements of sulfhydryl groups were made at 5 min and 90 min of incubation (Table 2).

The amount of –SH groups of pept_1545 after 5 min incubation with MPO/H_2_O_2_ decreased by about 20% and further did not change, suggesting cysteine oxidation by MPO. In addition to cysteine, pept_1545 contains tryptophan, another redox active amino acid. Similar to cysteine, free tryptophan is a poor peroxidase substrate for MPO. We sought to elucidate whether tryptophan of pept_1545 could be oxidized by H_2_O_2_-activated MPO by monitoring tryptophan intrinsic fluorescence which is known to decay upon tryptophan oxidative modification. Fluorescence measurements (λ_ex_ = 290 nm, λ_em_ = 310–450 nm) were made with 40 μM pept_1545 in the presence of MPO/H_2_O_2_ (0.4 μM heme, 50 μM H_2_O_2_) in 10 mM Na-phosphate buffer, pH 7.4. Neither inactive MPO nor MPO activated by H_2_O_2_ caused a significant decrease in the tryptophan fluorescence intensity.

Thus, our findings have shown that cysteine of pept_1545 can be oxidized by MPO. The assumption that cysteine, being incorporated in peptide, continues to be a poor substrate for MPO could explain the effects of pept_1545 on the absorbance spectra of MPO heme during peroxidase reaction (Figure 2C). At higher peptide concentrations of 200 μM and 250 μM, the reaction of cysteine with Compound I produced Compound II faster than the latter decayed, leading to Compound II accumulation (Figure 2C, the initial portion of black curve). As H_2_O_2_ was consumed with time, the slow conversion of the accumulated Compound II to ferric MPO could be accelerated by the reaction with cysteine (Figure 2C, right panel, the sharp decline in black curve). At lower concentrations of pept_1545, the reaction of cysteine with Compound I was too slow to provide Compound II accumulation, and the enzyme cycled. The accelerated Compound II reduction at lower pept_1545 concentrations, which was not preceded by a steady-state phase, could be attributed to cysteine as an additional electron donor. The accumulation of Compound II, which inhibited MPO, could be explained by limitation of the Compound II reduction by dissociation of pept_1545 from the peptide–MPO complex.

The results of studying the inhibition of MPO by using the antimicrobial peptide GRRRRSVQWCA demonstrated peptide binding in the peroxidase substrate pocket, enabling the peptide cysteine residue to be close to the heme group in the active site and interfere with reactive oxygen species within it, thus, inhibiting MPO activity [31]. Translocating the cysteine from position 10 to position 6 did not affect the inhibitory activity of the peptide. A stretch of arginine residues was shown to be involved in the binding. The positively charged character of arginine played a key role, since substitution of arginines to alanines abrogated an inhibitory effect, whereas replacement with lysines did not. In line with this is the presence of three positively charged residues -Lys-Arg-Lys- near the cysteine in pept_1545.

At this stage, the mechanism of the inhibitory effect of Hm-AMP8 is not clarified. Similar to pept_1545, Hm-AMP8 lost its sulfhydryl group upon incubation with H_2_O_2_-activated MPO, though at a lower rate as compared with pept_1545 (Table 2). As we discussed above, it can be speculated that tyrosine in Hm-AMP8 was the target for enzymatic oxidation, and the tyrosyl radicals formed reacted further with cysteine. The latter reaction could be limited by dissociation of Hm-AMP8 from the peptide–enzyme complex. This reaction could prevent dityrosine cross-links between the Hm-AMP8 molecules, which is consistent with negative fluorescence results on dityrosine formation in Hm-AMP8 incubated with MPO/H_2_O_2_. Perhaps the binding of Hm-AMP8 to MPO creates steric hindrance for substrate access to the active site, decreasing cycling of MPO.

### 3.5. Effects of CAMPs on MPO Peroxidase Activity

While an increased rate of enzyme turnover does not affect the yield of the final product, inhibition is a significant effect on enzyme activity. We determined the types and inhibition constants (K_i_) for inhibition of MPO peroxidase activity by Hm-AMP8 and pept_1545. The peroxidase activity was assayed by Amplex Red oxidation to fluorescent resorufin.

**Figure 5:**
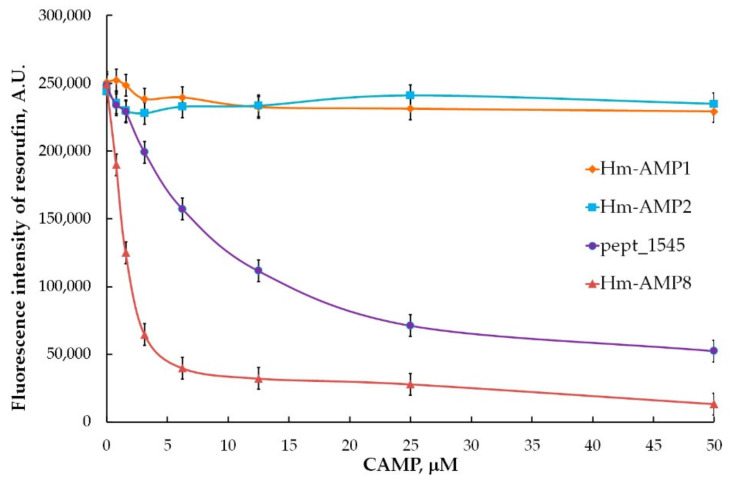
MPO peroxidase activity towards Amplex Red and H_2_O_2_ in the presence of the cationic antimicrobial peptides Hm-AMP1, Hm-AMP2, pept_1545, and Hm-AMP8 at 0.8–50 μM (peptide/heme molar ratio from 100 to 6250). The concentrations were as follows: 8 nM MPO heme, 50 μM Amplex Red, and 25 μM H_2_O_2_ in 50 mM K-phosphate buffer, pH 7.4.

**Figure 6:**
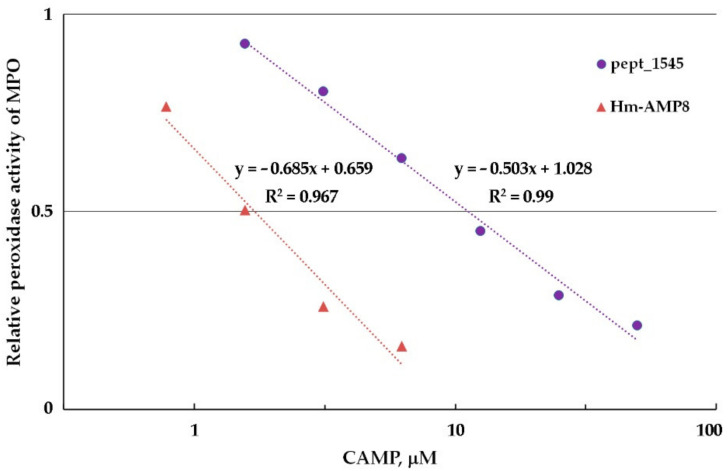
Graphical plot for determination of the half-inhibition concentration (IC_50_) of pept_1545 and Hm-AMP8 for MPO peroxidase activity. The concentrations were as follows: 8 nM MPO heme, 50 μM Amplex Red, and 25 μM H_2_O_2_ in 50 mM K-phosphate buffer, pH 7.4.

From the experiments with varying concentrations of H_2_O_2_ (0.01–200 μM) and Amplex Red (6.25–100 μM), the optimal concentrations of 25 μM H_2_O_2_ and 50 μM Amplex Red were chosen for further work. The 0.8–50 μM concentration range (corresponding to 100–6250 peptide molecules per heme) was used to determine the concentration dependence of the effect of CAMPs on peroxidase activity of MPO. As shown in Figure 5, Hm-AMP2 and Hm-AMP1 produced no inhibition. Pept_1545 and Hm-AMP8 dose-dependently inhibited MPO. These results are in line with the conclusions derived from the absorbance spectra in Figure 2. The IC_50_ values for Hm-AMP8 and pept_1545 were determined to be 1.7 μM and 11.2 μM, respectively (Figure 6).

**Figure 7:**
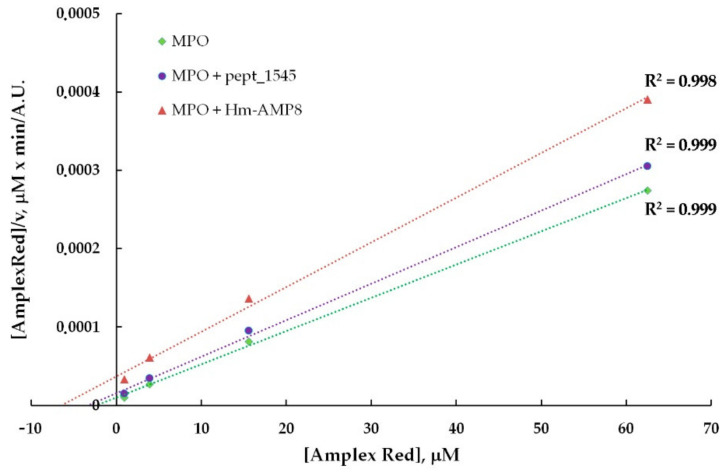
Hanes–Woolf plot illustrating the effect of 20 μM pept_1545 and 2 μM Hm-AMP8 on kinetics of MPO-catalyzed reaction of H_2_O_2_ with Amplex Red (*n* = 3). The concentrations were as follows: 8 nM MPO heme, 25 μM H_2_O_2_, and 1 μM– 62.5 μM Amplex Red in 50 mM K-phosphate buffer, pH 7.4.

**Figure 8:**
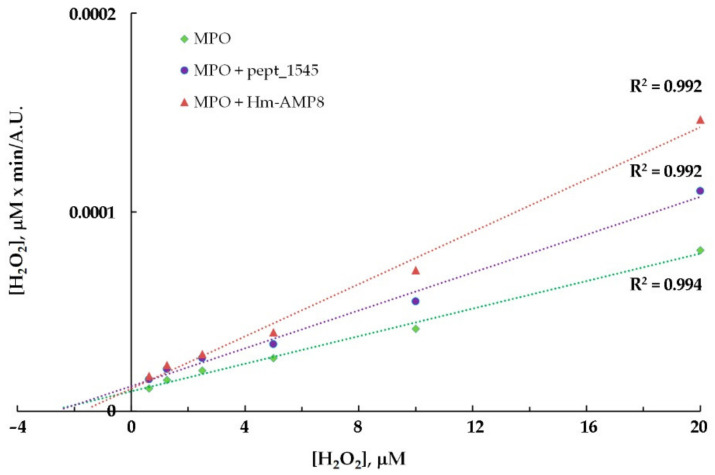
Hanes–Woolf plot illustrating the effect of 20 μM pept_1545 and 2 μM Hm-AMP8 on kinetics of MPO-catalyzed reaction of Amplex Red with H_2_O_2_ (*n* = 3). The concentrations were as follows: 8 nM MPO heme, 50 μM Amplex Red, and 0.625–20 μM H_2_O_2_ in 50 mM K-phosphate buffer, pH 7.4.

To identify the type of inhibition and to estimate K_i_, the kinetic data for MPO-catalyzed oxidation of Amplex Red in the presence of CAMPs were analyzed using the Hanes–Woolf linearizations (Figure 7 and Figure 8). The results of the inhibition analysis are presented in Table 3.

As seen from Table 3, pept_1545 inhibited MPO by competing with Amplex Red, but at the same time, it acted as an uncompetitive inhibitor, interacting with the MPO–H_2_O_2_ complex (probably, with Compound II), increasing MPO affinity for H_2_O_2_. Taken together, these facts suggest that pept_1545 can be a peroxidase substrate for MPO. Hm-AMP8 exhibited a more inhibitory effect, which was well marked at a concentration of 2 μM vs. 20 μM for pept_1545. K_i_, which reflects the dissociation constant for the interaction of Hm-AMP8 with MPO, which was 1.1 μM or 1.79 μM depending on the substrate. The latter value is almost the same as K_M_ for H_2_O_2_.

The mixed mechanism of inhibition, as determined by the kinetics of Amplex Red oxidation in the presence of pept_1545 and Hm-AMP8, may indicate several sites of interaction between the peptides and MPO. This, however, does not rule out that these peptides can be substrates for peroxidase activity of MPO. The uncompetitive inhibition implies that Hm-AMP8 and pept_1545 interact with the enzyme–substrate complex, and this mechanism for H_2_O_2_ and MPO means peptide interaction with Compounds I and II.

### 3.6. Effects of CAMPs on MPO Chlorinating Activity

**Figure 9:**
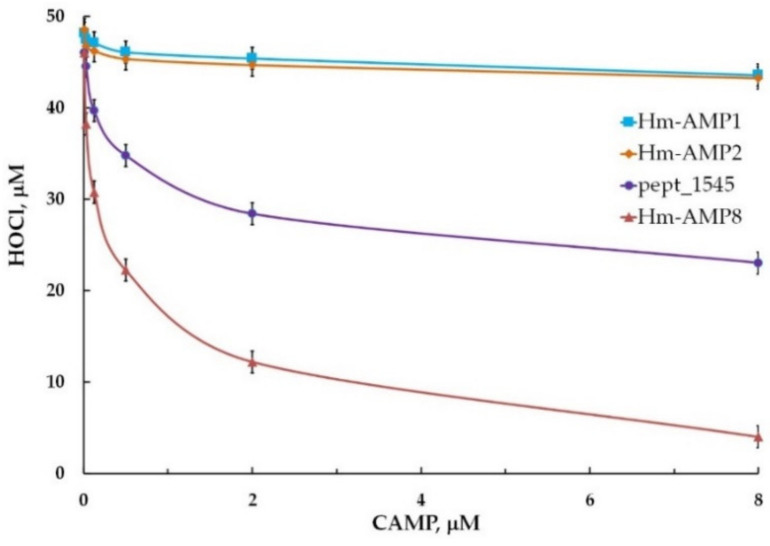
MPO chlorinating activity towards CB and H_2_O_2_ in the presence of the cationic antimicrobial peptides Hm-AMP1, Hm-AMP2, pept_1545, and Hm-AMP8 at 0.008–8 μM (peptide/heme molar ratios from 10 to 10,000). The concentrations were as follows: 0.8 nM MPO heme, 200 μM CB, 150 mM NaCl, and 50 μM H_2_O_2_ in 50 mM Na-acetate buffer, pH 5.5.

**Figure 10:**
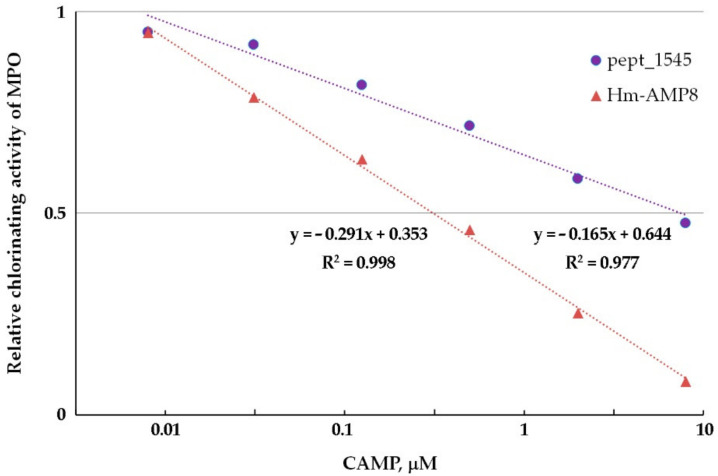
Graphical plot for determination of the half-inhibition concentrations of pept_1545 and Hm-AMP8 for MPO chlorinating activity. The concentrations were as follows: 0.8 nM MPO heme, 200 μM CB, 150 mM NaCl, and 50 μM H_2_O_2_ in 50 mM Na-acetate buffer, pH 5.5.

Chlorinating activity is a distinctive feature of MPO as compared with other peroxidases and it is an important property, enabling MPO to combat microbes. We tested whether the inhibitory effects of pept_1545 and Hm-AMP8 on peroxidase activity extend to MPO chlorinating activity. Chlorinating activity was measured using a fluorometric CB-based assay. To determine the concentration dependence of the effect of CAMPs on MPO chlorinating activity, the 0.008–8 μM concentration range (corresponding to 10–10,000 peptide molecules per heme) was used. Hm-AMP1 and Hm-AMP2 produced no significant effect, while pept_1545 and Hm-AMP8 inhibited MPO chlorinating activity similarly to peroxidase activity (Figure 9). The IC_50_ was 0.3 μM and 7.4 μM for Hm-AMP8 and pept_1545, respectively (Figure 10).

**Figure 11:**
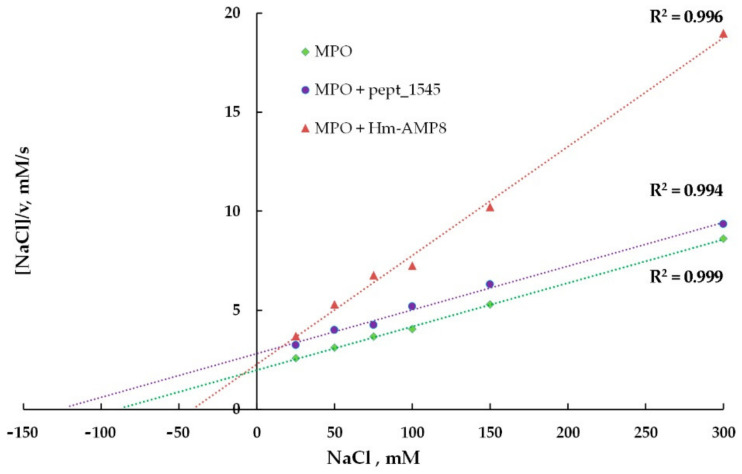
Hanes–Woolf plot illustrating the effects of 8 μM pept_1545 and 0.8 μM Hm-AMP8 on kinetics of MPO-catalyzed Cl^–^ oxidation (*n* = 3). The concentrations were as follows: 0.8 nM MPO heme, 200 μM CB, 25–300 mM NaCl, and 50 μM H_2_O_2_ in 50 mM Na-acetate buffer, pH 5.5.

**Figure 12:**
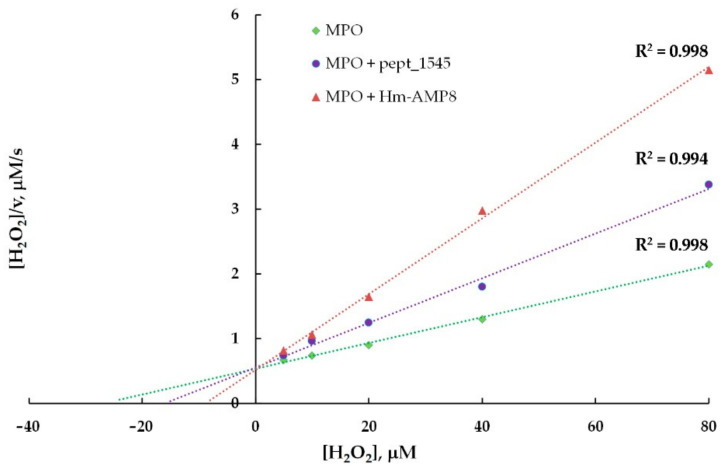
Hanes–Woolf plot illustrating the effects of 8 μM pept_1545 and 0.8 μM Hm-AMP8 on kinetics of MPO-catalyzed reaction of Cl^–^ with H_2_O_2_ (*n* = 3). The concentrations were as follows: 0.8 nM MPO heme, 200 μM CB, 150 mM NaCl, and 5–80 μM H_2_O_2_ in 50 mM Na-acetate buffer, pH 5.5.

To identify the types of inhibition produced by pept_1545 and Hm-AMP8 on MPO chlorinating activity and to estimate K_i_, the kinetic data for MPO-mediated oxidation of CB in the presence of peptides were analyzed using the Hanes–Woolf linearizations (Figure 11 and Figure 12). The results of the inhibition analysis are provided in Table 4.

As shown in Table 4, pept_1545 inhibited MPO chlorinating activity competitively towards the substrate Cl^–^ and uncompetitively towards the substrate H_2_O_2_. Hm-AMP8 exerted uncompetitive inhibition for any of the MPO substrates. The uncompetitive inhibition implies the interaction of peptides with the enzyme–substrate complex. For H_2_O_2_ and MPO, this means peptide interaction with Compounds I and II.

The effect of pept_1545 and Hm-AMP8 on MPO chlorinating activity can be attributed, on the one hand, to their cysteine. This amino acid is a preferred target for HOCl and N-chloramine. On the other hand, if these peptides act as peroxidase substrates, it is logical that they would compete with Cl^−^, since almost all peroxidase substrates of MPO are competitive inhibitors with respect to halide ions.

**Figure 13:**
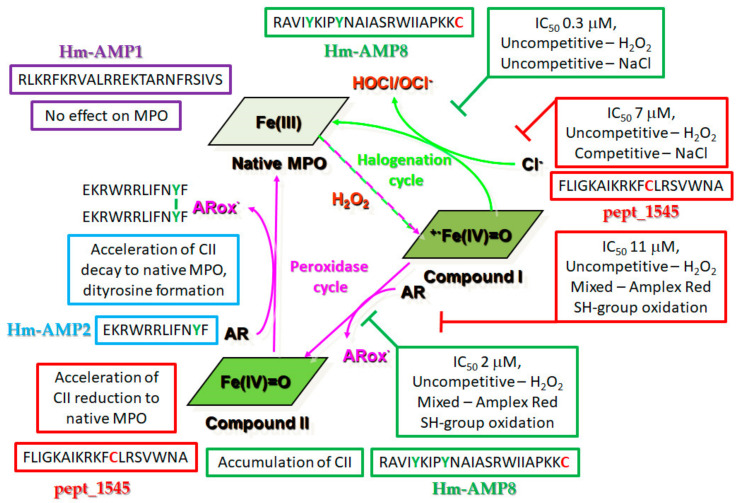
Proposed scheme for the effects of cationic antimicrobial peptides (CAMPs) under study on reactions of peroxidase and halogenation cycles of MPO. Native MPO existing in the ferric MPO-Fe(III) form reacts with hydrogen peroxide (H_2_O_2_) to form the redox intermediate Compound I. Compound I either oxidizes chloride (Cl^−^) to regenerate ferric MPO via the halogenation cycle (marked by green arrows) or will oxidize an organic substrate (AR) to a free radical (ARox), forming the redox intermediate Compound II, which can be reduced back to the native state via the peroxidation cycle (marked by pink arrows). Text in the box with red, green, blue, or purple border refers to pept_1545, Hm-AMP8, Hm-AMP2, and Hm-AMP1, respectively.

Below, a table is presented that summarizes the results of the research and a proposed scheme for interaction of CAMPs under study with redox intermediates of MPO in the peroxidase and halogenation cycles (Table 5 and Figure 13).

The results of this study indicate that among CAMPs, there are potential candidates to serve not only as antimicrobials but also as antioxidants with respect to aberrant MPO activity. Our findings on the ability of tyrosine- and cysteine-containing peptides to inhibit MPO activity are in agreement with those by [31,57].

## Figures and Tables

**Table 1:** Physical-chemical characteristics of cationic antimicrobial peptides (CAMPs) used in this study.

Peptide Name	Amino Acid Sequence	Mol. Mass, Da	Length	Net Charge (pH 7)	* Hydro- Phobicity	* Aliphatic Index	pI
Hm-AMP1	RLKRFKRVALRREKTARNFRSIVS	2988.61	24	+9	−0.95	81.25	12.9
pept_1545	FLIGKAIKRKFCLRSVWNA	2250.81	19	+5	0.33	107.8	11.6
Hm-AMP8	RAVIYKIPYNAIASRWIIAPKKC	2675.31	23	+5	0.21	114.78	10.6
Hm-AMP2	EKRWRRLIFNYF	1728.05	12	+3	−1.00	65	11.4

* Calculated using R package “Peptides” [40].

**Table 2:** Disappearance of free sulfhydryl groups in the mixture of cationic antimicrobial peptides (CAMPs) with MPO/H_2_O_2_. The amount of sulfhydryl groups is expressed as a percentage relative to the content in the mixture of CAMP and MPO with no added H_2_O_2_. The concentrations were 150 μM peptide, 1 μM MPO heme, and 100 μM H_2_O_2_ in 10 mM Na-phosphate buffer, pH 7.4. * *p* < 0.05.

Decline in Peptide’s Sulfhydryl Groups as a Result of Peptide Incubation with MPO/H_2_O_2_		
	5 min Incubation	90 min Incubation
Pept_1545	* 80 ± 4%	* 76 ± 5%
Hm-AMP8	93 ± 5%	* 79 ± 6%

**Table 3:** Pept_1545 and Hm-AMP8 inhibit MPO. Kinetic parameters for MPO peroxidase activity towards Amplex Red (upper panel) and H_2_O_2_ (lower panel), as measured by Amplex Red oxidation by MPO (8 nM heme) in the absence and presence of the peptides in 50 mM K-phosphate buffer, pH 7.4. The data presented were obtained using the Hanes–Woolf linearizations for different Amplex Red concentrations with 25 μM H_2_O_2_ and for different H_2_O_2_ concentrations with 50 μM Amplex Red.

Kinetic Parameters of MPO Peroxidase Activity towards Amplex Red			
	MPO	MPO/20 μM pept_1545	MPO/2 μM Hm-AMP8
Michaelis constant (K_M_), μM	2.4	3.4	6.5
Maximum reaction rate (V_max_), a. u.	235,917	214,729	175,664
Type of inhibition		mixed	mixed
Inhibition constant (K_i_), μM		48.3	1.1
**Kinetic parameters of MPO peroxidase activity towards H_2_O_2_**			
	**MPO**	**MPO/20** **μM pept_1545**	**MPO/2** **μM Hm-AMP8**
Michaelis constant (K_M_), μM	2.85	2.64	1.71
Maximum reaction rate (V_max_), a. u.	288,269	210,265	152,160
Type of inhibition		uncompetitive	uncompetitive
Inhibition constant (K_i_), μM		52.8	1.79

**Table 4:** Pept_1545 and Hm-AMP8 inhibit MPO. Kinetic parameters for MPO chlorinating activity towards chloride anion Cl^−^(upper panel) and H_2_O_2_ (lower panel), as measured by CB oxidation by MPO (0.8 nM MPO) in the absence and presence of the peptides in 50 mM Na-acetate buffer, pH 5.5. The data presented were obtained using the Hanes–Woolf linearizations for different NaCl concentrations with 50 μM H_2_O_2_ and for different H_2_O_2_ concentrations with 150 mM NaCl.

Kinetic Parameters of MPO Chlorinating Activity towards Cl^−^			
	MPO	MPO/8 μM pept_1545	MPO/0.8 μM Hm-AMP8
Michaelis constant (K_M_), μM	90	127	41.5
Maximum reaction rate (V_max_), s^−1^	45.4	45.2	18.2
Type of inhibition		competitive	uncompetitive
Inhibition constant (K_i_), μM		19.5	0.42
**Kinetic parameters of MPO chlorinating activity towards H_2_O_2_**			
	**MPO**	**MPO/8** **μM pept_1545**	**MPO/0.8** **μM Hm-AMP8**
Michaelis constant (K_M_), μM	27.0	16.0	8.9
Maximum reaction rate (V_max_), s^−1^	50.3	29.0	17.1
Type of inhibition		uncompetitive	uncompetitive
Inhibition constant (K_i_), μM		10.8	0.28

**Table 5:** Summary of findings table.

Interaction of CAMPs with MPO			
	H_2_O_2_-Induced Formation and Decay of MPO Compound II (CII). Oxidation of Peptide C and Y Residues	Inhibition of MPO Peroxidase Activity (Amplex Red Assay)	Inhibition of MPO Chlorinating Activity (CB Assay)
Hm-AMP8 RAVIYKIPYNAIASRWIIAPKKC	CII accumulation; –SH oxidation	IC_50_ = 2 μM Uncompetitive—H_2_O_2_ Mixed—Amplex Red	IC_50_ = 0.3 μM Uncompetitive—H_2_O_2_ Uncompetitive—Cl^−^
Pept_1545 FLIGKAIKRKFCLRSVWNA	Depending on concentration, acceleration of CII reduction to native MPO and CII accumulation; –SH oxidation	IC_50_ = 11 μM Uncompetitive—H_2_O_2_ Mixed—Amplex Red	IC_50_ = 7 μM Uncompetitive—H_2_O_2_ Competitive—Cl^−^
Hm-AMP2 EKRWRRLIFNYF	Acceleration of CII reduction to native MPO; diY formation	No effect	No effect
Hm-AMP1 RLKRFKRVALRREKTARNFRSIVS	No effect	No effect	No effect

cysteine (C red) and tyrosine (Y green).

## Data Availability

The data used to support the findings of this study are included within the article. Additional information may be obtained from the corresponding author upon request.

## Supplementary Materials

The following supporting information can be downloaded at: https://www.mdpi.com/article/10.3390/antiox11122419/s1, Table S1: Antibacterial efficacy of cationic antimicrobial peptides (CAMPs) used in this study. Figure S1: Comparative evaluation of hemolytic activity of the cationic antimicrobial peptides (CAMPs) used in this study.

## Sample Availability

The samples are not available from the authors.
