# Supplementary material for: Effects of Synthetic Short Cationic Antimicrobial Peptides on the Catalytic Activity of Myeloperoxidase, Reducing Its Oxidative Capacity
Source: Antioxidants (Basel). 2022 Dec 7;11(12):2419. doi: 10.3390/antiox11122419 (PMC9774438; doi:10.3390/antiox11122419)
Supplement: Supplementary file 1 [file antioxidants-11-02419-s001.zip › antioxidants-2084750-supplementary.pdf]

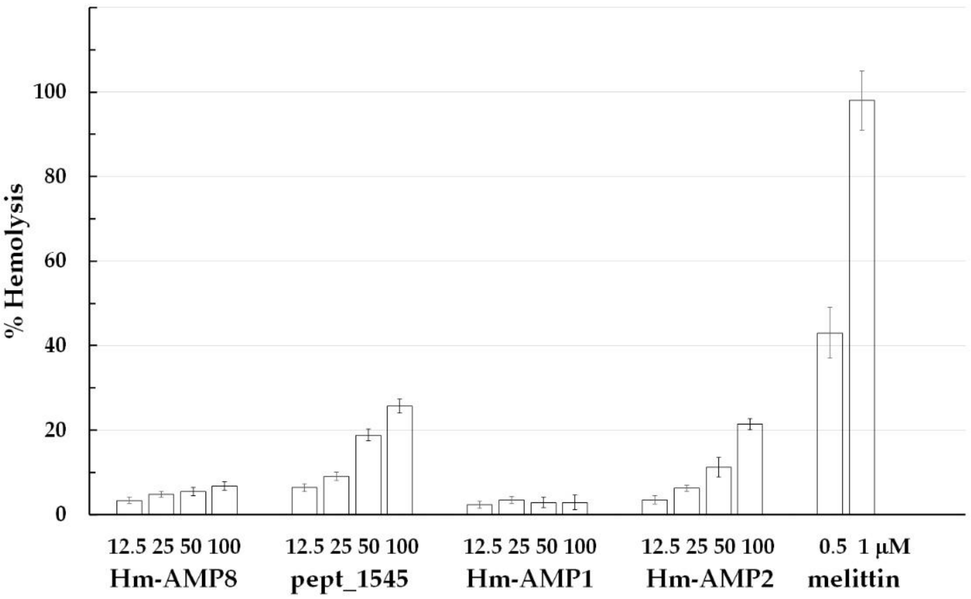

Figure S1. Comparative evaluation of hemolytic activity of the cationic antimicrobial peptides (CAMPs) Hm-AMP8, pept_1545, Hm-AMP1, and Hm-AMP2 using a hemolysis assay measuring hemoglobin released from damaged cells. In each group of bars, the final peptide concentration from left to right is 12.5 μM, 25 μM, 50 μM, and 100 μM. For melittin, the left bar – 0.5 μM, and the right bar – 1 μM. A working erythrocyte suspension (in PBS) was prepared so that its ten-fold dilution gave an optical density of 0.4 at 800 nm (about 30×10^6 cells/mL). Erythrocytes (125 μL) were added to the same volume of PBS containing the peptide. Cells with PBS served as negative control; cells with Triton X-100 served as positive control which was set to 100%. After 1-h incubation (with slight shaking) at 37°C, the samples were centrifuged at 1000 × g for 10 min, the supernatant was transferred to the wells of a microplate, and the absorbance of hemoglobin was measured by a Multiskan Ascent plate reader (Thermo Electron Corporation, Vantaa, Finland), with a test wavelength of 414 nm and a background wavelength of 690 nm. The hemolysis percentage was calculated using the following equation: Hemolysis (%) = {(sample absorbance – negative control) / (positive control – negative control)} × 100. Data shown are mean ± SD from three independent experiments using blood from three healthy volunteers, with triplicate samples in each experiment. All the volunteers gave informed consent to participate in the research.

As seen from Figure 1S, CAMPs used in this study were much less hemolytic than melittin, the well-known CAMP from bee venom. Melittin at 1 μM caused complete hemolysis, and it caused about 40% hemolysis at 0.5 μM, whereas none of CAMPs under study did not induce more than 30% hemolysis even at the highest concentration tested (100 μM). Note that the antibacterial capacity of certain CAMPs under study is almost the same or not much worse than that of melittin (Table 1S).

Table S1. Antibacterial efficacy of cationic antimicrobial peptides (CAMPs) used in this study

Minimum inhibitory concentration (MIC), μM					
	**Hm-AMP 1**	**pept_1545**	**Hm-AMP8**	**Hm-AMP2**	**melittin**
E. coli	>40	14	24	5	3
B. subtilis	11	7	12	9	3

The minimum inhibitory concentrations (MICs) for the Gram-negative bacterium *Escherichia coli* and Gram-positive bacterium *Bacillus subtilis* were determined earlier [20, 32]. The MICs of melittin (the well-known CAMP from bee venom) serving as a reference were also determined.
